# Fatty acid, tocopherol and squalene contents of Rosaceae seed oils

**DOI:** 10.1186/s40529-014-0048-4

**Published:** 2014-06-05

**Authors:** Bertrand Matthaus, Mehmet Musa Özcan

**Affiliations:** 1 Institute fur for Lipid Research, Federal Research Center for Nutrition and Food, Piusallee 76, Munster, 48165 Germany; 2 Department of Food Engineering, Faculty of Agriculture, Selcuk Universty, Konya, 42031 Turkey

**Keywords:** Rosaceae, Oil content, Fatty acid, Tocopherol, Squalen

## Abstract

**Background:**

The aim of current study is to establish the composition of these seeds belong to Rosaceae family with respect to fatty acid, tocopherol and squalene content.

**Results:**

The oil contents of seeds varied between 3.49 (*Cotoneaster bullatus*) to 46.15 g/100 g (*Prunus tenella*). The main fatty acids of seed oils were oleic (6.50 - 67.11 %), linoleic (22.08 - 68.62 %) and 20:1n-7 (0.10 - 61.59 %). As observed, the oils of seed were rich in linoleic and oleic acids. Total tocopherol contents ranged between 7.06 mg/100 g (*Prunus tenella*) to 165.74 mg/100 g (*Potentilla glandulosa* ssp. *pseudorupestris*). The major tocopherols were γ-tocopherol, ranging from 2.08 mg/100 g to 106.01 mg/100 g; α-tocopherol ranging from 2.86 mg100 g to 74.26 mg/100 g and δ-tocopherol ranging used in this experiment were found between 0.02 mg/100 g (*Alchemilla caucasica*) to o.29 mg/100 g (*Cotoneaster simonsii*).

**Conclusions:**

These results show that Rosaceae seed oils can be a potential saurce of valuable oil which might be useful for the evaluation of dietary information in important food crops and other industrial applications.

## Background

Some seed oils are already used for several purposes: blending higly saturated edible oils to provide new oils with modified nutritional values, as ingredients in paint and varnish formulations, surface coating and oleo-chemicals and as oils for cosmetic purposes (Helmy [1990]). Several species of Rosaceae presently have great commercial value as oil crops, e.g. *Aronia melanocarpa* L., *Rosa canina*, severeal and *Rubus* spp (Pourrat and Carnat [1981]; Johansson et al. [1997]; Xu et al. [2006]; Oh et al. [2007]). Plant seeds are important sources of oils of nutritional, industrial and pharmaceutical importance. No oil from any single source has been found to be suitable for all purposes because oils from different sources generally differ in their fatty acid composition. The fatty acid composition of the endogenous fats plays an important role in determining shelf life, nutrition and flavor of food products (Gao and Mazza [1995]). The study of oil seeds for their minor constituents is useful in order that both the oil and its minor constituents be used effectively (Ramadan and Mörsel [2002]). Tocopherols and squalene are components present in the unsaponifiable lipid fraction of foods. α- Tocopherol, the most common form of vitamin E present in nature, is the most biologically active (Bjorneboe et al. [1990]), and is preferentially retained in large quantities and transported to body components (Traber et al. [1990]; Ching and Mohamed [2001]). Konings et al. ([1996])) developed a HPLC method for determination of tocopherols and tocotrienols in margarine, infant foods and vegetables. The main biochemical function of the tocopherols is believed to be the protection of polyunsaturated fatty acids aganist peroxidation (Beringer and Dompert [1976]; Kamel-Eldin and Andersson [1997]). They have vitamin E properties and display antioxidant activity, which protect the body tissues aganist the damaging effects caused by the free radicals that results from many normal metabolic functions (Lopez Ortiz et al. [2006]). The major fatty acids composing the oils were linoleic (41-70%), linolenic (13-36%), and oleic (11-19%) acids (Pourrat and Carnat [1981]; Johansson et al. [1997]; Oomah et al. [2000]; Bushman et al. [2004]). At the same time, tocopherols, the major vitamin of vitamin E are fat-soluble antioxidants that function as scavengers of lipid peroxyl radicals (Ryan et al. [2007]).

Squalene, a 30 carbon isoprenoid, is a key intermediate in cholesterol biosynthesis and is abundant in shark liver oil and olive oil (Ryan et al. [2007]). More recently, squalene has been shown to act as an antidote to reduce accidental drug-induced toxicities (Aguilera et al. [2005]; Senthilkumar et al. [2006]; Ryan et al. [2007]). The protective effect of squalene may be attributed to its ability to serve as an antioxidant (Ryan et al. [2007]). To achieve the most economical and efficient utilization of these seeds, more information on the varieties, properties and composition is required. Therefore, the present study attempted to establish the composition of these seeds belong to Rosaceae family with respect to fatty acid, tocopherol and squalene content.

## Methods

### Seeds

About 26 variety of plants belong to Rosaceae family were collected from plants growing in Botanical Garden of Germany (*Alchemilla caucasica, Cotoneaster bullatus, Cotoneaster dielsianus, Cotoneaster francheti, Cotoneaster moupinensis, Cotoneaster simonsii, Dryas drummondii, Exochorda racemosa, Geum elatum,Geum magellanicum, Geum pyrenaicum, Potentilla alchimilloides, Potentilla ambigua, Potentilla argyrophylla var.leucochroa, Potentilla atrosanguinea, Potentilla aurea, Potentilla glandulosa, Potentilla grammopetala, Potentilla hippiana, Potentilla pyrenaica, Potentilla speciosa, Potentilla tridentate, Potentilla visianii, Prinsepia uniflora, Prunus tenella* and *Rosa palustris*) in August, 2007 year. The seeds were cleaned in air screen cleaner to remove immature and broken seeds, dried by air condition. The seeds were stored in paper bags at +4°C temperature.

### Reagents

Petroleum ether (40–60°C) was of analytical grade (>98%; Merck, Darmstadt, Germany). Heptane and tert-butyl methyl ether were of HPLC grade (Merck, Darmstadt, Germany). Tocopherol and tocotrienol standard compounds were purchased from CalBiochem (Darmstadt, Germany).

### Oil content

The oil content was determined according to the method ISO 659:1998 (ISO,1998). About 2 g of the seeds were ground in a ball mill and extracted with petroleum ether in a Twisselmann apparatus for 6 h. The solvent was removed by a rotary evaporator at 40°C and 25 Torr. The oil was dried by a stream of nitrogen and stored at – 20°C until used.

### Fatty acid composition

The fatty acid composition was determined following the ISO standard ISO 5509:2000 (ISO 2000). In brief, one drop of the oil was dissolved in 1 mL of *n*-heptane, 50 μg of sodium methylate was added, and the closed tube was agitated vigorously for 1 min at room temperature. After addition of 100 μL of water, the tube was centrifuged at 4500 g for 10 min and the lower aqueous phase was removed. Then 50 μL of HCl (1 mol with methyl orange) was added, the solution was shortly mixed, and the lower aqueous phase was rejected. About 20 mg of sodium hydrogen sulphate (monohydrate, extra pure; Merck, Darmstadt, Germany) was added, and after centrifugation at 4500 g for 10 min, the top n-heptane phase was transferred to a vial and injected in a Varian 5890 gas chromotograph with a capillary column, CP-Sil 88 (100 m long, 0.25 mm ID, film thickness 0.2 μm). The temperature program was as follows: from 155°C; heated to 220°C (1.5°C/min), 10 min isotherm; injector 250°C, detector 250°C; carrier gas 36 cm/s hydrogen; split ratio 1:50; detector gas 30 mL/min hydrogen; 300 mL/min air and 30 mL/min nitrogen; manual injection volume less than 1 μL. The peak areas were computed by the integration software, and percentages of fatty acid methyl esters (FAME) were obtained as weight percent by direct internal normalization.

### Tocopherols

For determination of tocopherols, a solution of 250 mg of oil in 25 mL of n-heptane was directly used for the HPLC. The HPLC analysis was conducted using a Merck-Hitachi low-pressure gradient system, fitted with a L-6000 pump, a Merck-Hitachi F-1000 fluorescence sp ectrophotometer (detector wavelengths for excitation 295 nm, for emission 330 nm), and a D-2500 integration system. The samples in the amount of 20 μL were injected by a Merck 655-A40 autosampler onto a Diol phase HPLC column 25 cm × 4.6 mmID (Merck, Darmstadt, Germany) used with a flow rate of 1.3 mL/min. The mobile phase used was n-heptane/tert-butyl methyl ether (99 + 1, v/v).

## Results and discussion

Percentages of the lipidic fraction of the 26 plant seeds belong to Rosaceae familya re given in Table 1. Oil contents of samples changed between 3.49 g/100g and 46.15 g/100g. On average terms, seeds contained 19.45%. However, due to the economical value of oil content, they are both valuable as raw material for oil extraction. The authors found numerous references in the literature composition of the lipidic fraction for some Rosaceae seeds. Studies based on varieties give results that ranged between 9.0 and 23% (Pourrat and Carnat [1981]; Johansson et al. [1997]; Zlatanov [1999]; Oomah et al. [2000]; Bushman et al. [2004]; Oh et al. [2007]).

**Table 1:** **Oil contents of some Rosaceae seeds**

Samples	Oil contents (%)
*Alchemilla caucasica*	25.99
*Cotoneaster bullatus*	3.49
*Cotoneaster dielsianus*	7.56
*Cotoneaster francheti*	5.22
*Cotoneaster moupinensis*	5.29
*Cotoneaster simonsii*	3.91
*Dryas drummondii*	21.82
*Exochorda racemosa*	19.56
*Geum elatum*	17.38
*Geum magellanicum*	19.04
*Geum pyrenaicum*	14.53
*Potentilla alchimilloides*	20.23
*Potentilla ambigua*	9.71
*Potentilla argyrophylla var.leucochroa*	17.48
*Potentilla atrosanguinea*	23.81
*Potentilla aurea*	28.93
*Potentilla glandulosa*	28.95
*Potentilla grammopetala*	19.01
*Potentilla hippiana*	28.60
*Potentilla pyrenaica*	23.92
*Potentilla speciosa*	15.09
*Potentilla tridentate*	18.41
*Potentilla visianii*	23.80
*Prinsepia uniflora*	41.82
*Prunus tenella*	46.15
*Rosa palustris*	16.06

The most abundant fatty acids in seed oils were oleic, linoleic and 20:1n-7 acids, accounting for 96.63 to 99.80% in seed oils. The oils extracted from Rosaceae seeds were composed of 3.25-9.17% palmitic, 1.19-4.27% stearic, 6.50-67.11% oleic, 22.08-68.62% linoleic and 0.10-61.59% eicosenoic acids (Table 2). The proportion of linoleic acid in the seed oil of *Exochorda racemosa* was higher than that in the seed oil of *Potentilla hippiana*. This proportion was also higher than those of in other some seed oils (Pourrat and Carnat [1981]; Bushman et al. [2004]; Matthaus and Ozcan [2005]; Oh et al. [2007]). The erucic acid species of this genus contain from the linolenic acid (Table 2). As can be observed, the oils of all seed oils used in this experiment had higher linoleic acid content than those of other fatty acids. On the other hand, oleic acid contents of seed oils varied between 6.5% (*Potentilla argyrophylla* var. *leucochroa*) to 67.11% (*Prunus tenella*).

**Table 2:** **Fatty acid compositions of Rosaceae seed oils (%)**

Samples	Fatty acids								
	Palmitic	Palmitoleic	Stearic	Oleic	Vaccenic	Linoleic	Linolenic	Eicosenoic	20:1n-7
*Alchemilla caucasica*	4.15	0.07	1.96	10.54	0.31	31.29	-	0.24	50.29
*Cotoneaster bullatus*	7.89	0.29	1.45	20.57	1.26	60.04	3.30	-	0.46
*Cotoneaster dielsianus*	9.17	0.13	4.27	29.81	0.28	51.89	0.67	-	0.42
*Cotoneaster francheti*	8.77	0.16	1.37	16.67	0.49	66.60	0.77	-	0.62
*Cotoneaster moupinensis*	6.66	0.21	1.48	26.59	0.44	59.85	0.88	-	0.53
*Cotoneaster simonsii*	6.81	0.25	1.19	19.52	0.49	64.64	1.47	-	0.94
*Dryas drummondii*	5.92	0.30	1.73	22.72	0.40	56.82	0.05	1.31	6.20
*Exochorda racemosa*	6.84	0.18	2.50	18.54	0.43	68.62	0.47	-	0.16
*Geum elatum*	8.19	0.17	2.06	20.30	0.40	39.25	0.06	0.06	27.20
*Geum magellanicum*	4.73	0.06	2.07	17.62	0.39	29.69	0.03	0.13	43.23
*Geum pyrenaicum*	6.06	0.18	3.43	34.45	0.51	22.61	0.07	0.08	30.28
*Potentilla alchimilloides*	6.09	0.09	2.44	7.89	0.40	26.74	-	0.16	55.02
*Potentilla ambigua*	5.65	0.18	0.98	13.13	0.57	22.67	0.37	0.12	52.70
*Potentilla argyrophylla*	4.46	0.15	1.44	6.50	0.41	28.04	-	0.63	57.25
*Potentilla atrosanguinea*	4.20	0.13	1.44	11.31	0.43	32.84	-	0.14	48.59
*Potentilla aurea*	5.16	0.13	1.59	10.91	0.35	26.17	-	0.84	53.78
*Potentilla glandulosa*	4.37	0.22	1.22	13.41	0.51	25.91	-	0.82	52.20
*Potentilla grammopetala*	5.32	0.09	2.00	9.77	0.36	27.89	-	0.52	52.91
*Potentilla hippiana*	4.64	0.12	1.40	10.80	0.70	22.08	0.03	0.16	57.89
*Potentilla pyrenaica*	4.93	0.08	1.99	12.90	0.29	29.65	-	0.86	48.28
*Potentilla speciosa*	7.81	0.13	2.07	19.21	0.55	26.73	-	0.11	40.94
*Potentilla tridentate*	4.30	0.13	1.88	14.13	0.39	40.85	-	0.60	36.53
*Potentilla visianii*	3.25	0.05	1.55	8.14	0.33	23.27	-	0.70	61.59
*Prinsepia uniflora*	5.20	0.17	1.75	28.22	0.41	60.92	0.34	-	0.44
*Prunus tenella*	3.48	0.23	-	67.11	-	26.76	0.16	-	0.10
*Rosa palustris*	5.04	0.19	1.74	15.77	0.56	27.56	0.15	0.83	45.37

Nutritionally unfavorable is the high content of saturated fatty acids, consisting of palmitic acid, which amounted to between 3.25% (*Potentilla visianii*) to 9.17% (*Cotoneaster dielsianus*), and stearic acid, which was found in a very small range between 1.19% (*Cotoneaster simonsii*) to 4.27% (*Cotoneaster dielsianus*). However, Rosaceae seed oil used in this experiment contain more linoleic and 20:1 n-7 (except for a few seed oils) acids and less stearic and palmitic acids. Also, the oils of some seed, contained a high propertion of 20:1 n-7. The main fatty acids in bramble seed oils are C18:2n −6 (51.0-66.1%), C18:3n-3 (9.70-35.6%), C18:1n-9 (9.85-16.3) and C16:0 (2.01-5.73%) (Xu et al. [2006]). In general, high amounts of linoleic acid are unsuitable for oil-food products due to its instability and reversion of flavor associated with autoxidation (Green [1986]; Singh et al. [1998]). So, these seed oils may be a suitable oil seed crop for the various industry due to its very low content of linolenic and high content of linoleic acid (Singh et al. [1998]). Those observations are in agreement with the data reported earlier about the fatty acid composition of some seed oils (Tiscornia et al. [1976]; Zlatanov et al. [1997]; Zlatanov [1999]).

Most plants deriveted foods contain low to moderate levels of vitamin E activity. However, oving to the abundance of plant-derived foods in our diets, they provide a significant and consistent source of vitamin E (Eitenmiller and Lee [2004]; Ryan et al. [2007]). The tocopherol contents of seed oils researched in present study are listed in Table 3. All the seeds analysed exhibited differences in their tocopherol contents and the differences were found. The major tocopherol was γ-tocopherol in all the varieties of Rosaceae seed oils, which was higher in *Potentilla glandulosa* (106.01 mg/100 g) than in *Cotoneaster simonnsii* (2.08 mg/100 g). The contents of α-tocopherol in seed oil of *Cotoneaster simonsii* (74.26 mg/100 g) were about 26x that of *Prunus tenella* (2.86 mg/100 g), and the content of tocopherol in *Potentilla aurea* seed oil (73.59 mg/100 g) was also hipher than that in *Alchemilla caucasica* (0.60 mg/100 g) oil. The total tocopherol in *Potentilla glandulosa* seed oil (165.74 mg/100 g) was higher than that in *Prunus tenella* (7.06 mg/100 g). Our results are higher than those of other authors (α-tocopherol and β + γ-tocopherol) (Ryan et al. [2007]). The major tocopherol in all bramble seed oils of 10 varieties was γ-tocopherol. The composition (mg/100 g) was as follows: α-tocopherol 7.65-52.6, γ-tocopherol 46.9-106, δ-tocopherol 3.1-9.50, and the active vitamin E 15.9-61.5 among the varieties (Xu et al. [2006]). In the tocopherol fraction (55.5 mg/kg in chokeberry oil, 249.6 mg/kg in black currant oil and 89.4 mg/kg in rose hip oil), α-tocopherol predominated in chokeberry oil (70.6 mg/kg). γ-Tocopherol was the main component in black currant oil (55.4 mg/kg) and rose hip oil (71.0 mg/kg) (Zlatanov [1999]). The content of tocopherols is 360 mg/100 g in the hexane extract oil of raspberry seed and the main component is the isomer (Oomah et al. [2000]). In cold- pressed raspberry seed oil, the total tocopherols are 88.9 mg/100 g (Parry et al. [2005]). So, because of the nutritional and antioxidant properties of tocopherols, Rosaceae seed oils should be taken into account. As a healthy product, the fatty acids and tocopherols in the seed oil are the major components (Oomah et al. [2000]).

**Table 3:** **Tocopherol contents of some Rosaceae seed oils (mg/100 g)**

	α	α-T3	β-T	γ-T	β-T3	P8	γ-T3	Δ-T	Δ-T3	Sum
*Alchemilla caucasica*	6.17	5.64	0.00	15.90	0.00	0.00	0.00	0.60	0.00	28.31
*Cotoneaster bullatus*	53.56	56.84	0.00	285.49	0.00	0.00	0.00	0.00	0.00	138.88
*Cotoneaster dielsianus*	21.87	21.29	0,00	11,77	0,00	0,00	0,00	0,68	0,00	55,61
*Cotoneaster francheti*	34.10	29.11	1,72	28,38	0,00	0,00	0,00	3,97	0,00	97,27
*Cotoneaster moupinensis*	27.33	0.00	7,14	0,00	0,00	0,00	0,00	0,00	0,00	34,47
*Cotoneaster simonsii*	74.26	0,00	74,72	2,08	0,00	5,10	0,00	1,57	0,00	157,73
*Dryas drummondii*	10.47	0,00	0,37	13,68	0,00	0,42	0,00	0,69	0,00	25,63
*Exochorda racemosa*	25,48	0,00	0,00	87,09	0,00	0,00	0,00	4,21	0,00	116,78
*Geum elatum*	42,92	10,03	3,43	12,40	0,00	0,22	0,19	1,70	0,00	70,89
*Geum magellanicum*	23,16	7,75	2,71	41,94	0,00	0,65	0,51	5,21	0,00	81,93
*Geum pyrenaicum*	32,86	11,81	0,82	24,59	0,00	0,54	0,37	0,99	0,00	71,97
*Potentilla alchimilloides*	11,67	10,17	0,50	27,48	0,00	0,36	0,39	4,98	0,00	55,55
*Potentilla ambigua*	20,43	17,02	0,41	58,10	0,00	0,00	0,00	2,69	0,00	98,64
*Potentilla argyrophylla*	12,51	10,14	1,21	48,55	0,00	0,23	0,27	44,56	0,00	117,45
*Potentilla atrosanguinea*	9,88	7,75	99,00	37,11	0,00	0,95	0,47	25,33	0,00	82,48
*Potentilla aurea*	7,58	5,54	1,54	39,38	0,00	0,29	0,38	73,59	0,00	128,30
*Potentilla glandulasa*	5,89	0,00	0,28	106,01	0,00	0,46	0,16	52,94	0,00	165,74
*Potentilla grammopetala*	8,06	0,00	0,41	33,42	0,00	0,29	0,47	3,96	0,00	46,61
*Potentilla hippiana*	5,76	0,00	0,00	58,16	0,00	0,45	0,24	41,28	0,00	105,89
*Potentilla pyrenaica*	10,13	8,05	1,41	15,04	0,00	0,30	0,25	68,30	0,53	104,01
*Potentilla speciosa*	22,75	13,34	0,49	50,77	0,00	0,00	0,51	2,85	0,00	90,71
*Potentilla tridentata*	10,96	9,97	0,18	21,83	0,00	0,42	0,44	0,83	0,00	44,63
*Potentilla visianii*	9,17	7,50	0,39	55,99	0,00	0,31	0,25	9,28	0,00	82,89
*Prinsepia uniflora*	6,11	0,00	0,00	39,79	0,00	0,00	0,29	2,85	0,00	49,04
*Prunus tenella*	2,86	0,00	0,16	2,57	0,00	0,00	0,70	0,78	0,00	7.06
*Rosa palustris*	11.55	0.00	0.60	43.05	0.00	0.59	0.26	1.90	0.72	58.67

Squalene was determined in some Rosaceae seed oils employed in the present study; levels of squalene were found between 0.02 mg/100 g (*Alchemilla caucasica*) and 0.29 mg/100 g (*Cotoneaster simonsii*) (Table 4). Squalene, a biosynthetic precurson to all steroids both in plant and animal cells, also exists with phytosterols and tocopherols in the unsaponifiable fraction of foods. There is an obvious scarcity of data on squalene content in foods (Ryan et al. [2007]).

**Table 4:** **Squalene contents of some Rosaceae seeds**

Samples	Concentrations (%)
*Alchemilla caucasica*	0.02
*Cotoneaster bullatus*	0.22
*Cotoneaster dielsianus*	0.07
*Cotoneaster francheti*	0.13
*Cotoneaster moupinensis*	0.16
*Cotoneaster simonsii*	0.29
*Dryas drummondii*	0.09
*Exochorda racemosa*	0.14
*Geum elatum*	0.09
*Geum magellanicum*	0.05
*Geum pyrenaicum*	0.13
*Potentilla alchimilloides*	0.05
*Potentilla ambigua*	0.12
*Potentilla argyrophylla var.leucochroa*	0.05
*Potentilla atrosanguinea*	0.04
*Potentilla aurea*	0.04
*Potentilla glandulosa*	0.05
*Potentilla grammopetala*	0.07
*Potentilla hippiana*	0.03
*Potentilla pyrenaica*	0.05
*Potentilla speciosa*	0.07
*Potentilla tridentate*	0.05
*Potentilla visianii*	0.06
*Prinsepia uniflora*	0.04
*Prunus tenella*	-
*Rosa palustris*	0.10

In the tocopherol fraction (55.5 mg/kg in chokeberry oil, 249.6 mg/kg in black currant oil and 89.4 mg/kg in rose hip oil), α-tocopherol predominated in chokeberry oil (70.6 mg/kg). Γ-tocopherol was the main component in black currant oil (55,4 mg/kg) and rose hip oil (71.0 mg/kg) (Zlatanov [1999]).

Among plant foods, amaranth, a pseudo cereal grain, contains relatively high amounts of squalene, approximately 132 mg/100 g to 424 mg/100 g (Berganza et al. [2003]). Another exceptionally rich source of squalene is olive oil, which is reported to contain 2000 to 7000 μg/g oil (Liu et al. [1976]). In another study, Ryan et al. ([2007])) identified 58.4 and 89.0 mg/100 g squalene in quinoa and pumpkin seed, respectively. The contents of other samples ranged between 0.2 barley to 8.8 mg/100 (Millet). As a result, the squalene content of Rosaceae seed oils employed in the present study is lower than that of the squalene content reported for poppy, mustard pumpkin, sesame, millet, quinoa, spelt, lentils, peas and especially olive (Liu et al. [1976]; Ryan et al. [2007]). In addition, several experimental studies demonstrated the detoxifying activities of squalene against a wide range of chemicals such as arsenic, hexacholorobengene and Phenobarbital (Kamimara et al. [1992]; Fan et al. [1996]; Ryan et al. [2007]).

## Conclusion

The oil contents of seeds varied between 3.49 (*Cotoneaster bullatus*) to 46.15 g/100 g (*Prunus tenella*). The main fatty acids in seed oils were oleic (6.50-67.11%) and linoleic (22.08-68.62%). The concentrations of total tocopherol ranged between 7.06 mg/100 g (*Prunus tenella*) to 165.74 mg/100 g (*Potentilla glandulosa* ssp. *pseudorupestris*). Squalene was determined in some Rosaceae seed oils employed in the present study; levels of squalene were notably low between 0.02 mg/100 (*Alchemilla caucasica*) to 0.29 mg/100 g (*Cotoneaster simonsii*). The present study indicates that some Rosaceae seeds are good natural sources of oil. In addition, fatty acids, tocopherol and squalene in particular seem to have a very important effect on health.
